# Effectiveness of Virtual Reality Simulation for Tracheostomy Education Among Healthcare Professionals

**DOI:** 10.7759/cureus.97083

**Published:** 2025-11-17

**Authors:** Haleema Siddique, Ali Abbas, Ayeshah Abdul-Hamid

**Affiliations:** 1 Otolaryngology, Oxford University Hospitals NHS Foundation Trust, Oxford, GBR

**Keywords:** airway, airway obstruction, endoscopy, laryngology, laryngomalacia, medical education, paediatric airways, stridor, surgical techniques

## Abstract

Objective: The objective of this study is to evaluate the effectiveness of virtual reality (VR) simulation in tracheostomy teaching for healthcare professionals and its impact on short- and medium-term knowledge retention.

Method: A prospective educational study was conducted among 26 healthcare professionals (17 VR; 9 non-VR). Both groups received standard face-to-face tracheostomy teaching, while the VR group additionally completed a 40-minute hybrid simulation using Goggleminds™ software based on the National Tracheostomy Safety Project algorithm. Knowledge was assessed using an eight-item multiple-choice questionnaire immediately after training (Day 0) and after 30 days (Day 30). Data were analysed using independent-samples *t*-tests and Mann-Whitney *U* tests to confirm robustness.

Results: Participants in the VR group achieved significantly higher mean scores immediately after training (7.24 ± 0.75) compared with the non-VR group (6.33 ± 0.87; *p* = 0.01). At 30 days, the VR group demonstrated superior knowledge retention (6.88 ± 0.93) compared with the non-VR group (4.67 ± 1.00; *p* < 0.001). These findings indicate that VR-based tracheostomy simulation enhances both learning and retention of knowledge compared with conventional teaching.

Conclusion: VR-based tracheostomy simulation significantly improved knowledge acquisition and 30-day retention among healthcare professionals. Integrating VR into tracheostomy education may help to standardise training, improve confidence, and enhance patient safety in emergency airway management.

## Introduction

Tracheostomy management is a critical skill across multiple medical and surgical specialities, including otolaryngology, oral and maxillofacial surgery, anaesthetics, and intensive care. The National Confidential Enquiry into Patient Outcome and Death (NCEPOD) reported that approximately 12,000 tracheostomies are performed annually in the United Kingdom, with over one-fifth carried out for upper airway obstruction [1]. Despite the frequency of the procedure, post-tracheostomy complications remain common in both intensive care and ward environments, with accidental decannulation being one of the most serious and recurrent issues [1].

The same report identified variability in training and emergency preparedness for tracheostomy management across healthcare institutions, with only 80.6% of acute hospital trusts confirming the presence of a formal policy for emergency tracheostomy care [1]. This highlights a continuing need for consistent and effective training to ensure rapid, coordinated management of tracheostomy-related emergencies.

Simulation-based education has been increasingly adopted to address such training gaps. The Chief Medical Officer’s report Safer Medical Practice advocated for the wider use of simulation in clinical training within the NHS, recognising its potential to enhance competence and patient safety [2]. Simulation provides a safe environment in which healthcare professionals can practise high-risk or low-frequency procedures, refine psychomotor skills, and improve decision-making without compromising patient safety [3].

Recent technological advances have enabled the use of virtual reality (VR) in medical education. VR simulation provides an immersive, interactive environment that integrates visual, auditory, and haptic feedback, allowing users to engage in realistic clinical scenarios [4]. Within surgical disciplines, VR has shown promise in improving procedural accuracy, confidence, and knowledge retention [5,6]. However, its application in tracheostomy training remains limited, and data on its educational impact in this specific context are sparse.

The present study aimed to evaluate the effectiveness of VR-based tracheostomy simulation compared with traditional teaching methods in improving immediate knowledge acquisition and medium-term retention among healthcare professionals. The primary objective was to assess whether participants exposed to VR simulation achieved higher knowledge scores immediately after training and at 30 days post-intervention than those receiving conventional teaching. The secondary objective was to explore participant feedback regarding the acceptability, comfort, and perceived educational value of the VR platform and to consider the feasibility of incorporating VR into future regional training programmes.

## Materials and methods

Study design and ethics

A prospective, single-centre educational study was conducted during a regional tracheostomy study day in the United Kingdom. As this project evaluated an educational intervention among healthcare professionals and did not involve patients, patient data, or identifiable personal information, formal ethical approval was not required according to institutional policy. All participants were informed about the purpose of the study and provided verbal consent to participate and for the use of anonymised data for publication.

Participants and setting

Twenty-six healthcare professionals participated in the study, including otolaryngology and oral and maxillofacial speciality registrars (ST6 and above). Participants were allocated non-randomly based on workshop scheduling: 17 were assigned to the VR group and nine to the non-VR (control) group.

Intervention

Both groups received standardised face-to-face teaching on tracheostomy care delivered as part of the study day programme. The VR group additionally completed a 40-minute hybrid simulation session using Goggleminds™ (Cardiff, UK) software, which followed the National Tracheostomy Safety Project (NTSP) emergency algorithm [4]. Each VR session consisted of a 10-minute pre-brief, a 20-minute interactive simulation, and a 10-minute debrief.

The hybrid agenda for the study day is presented in Table 1 (all times in GMT). The VR module was incorporated alongside other viva stations, ensuring no extension of the scheduled programme duration. The module itself is a step-by-step, user-friendly software programme that can be set up independently by learners. 

**Table 1:** Agenda for the Regional Tracheostomy Study Day (All times in Greenwich Mean Time, GMT)

Hybrid	09:30-09:40	Introductions
	09:40-10:10	The Surgical Overview
	10:10-10:30	Types of tracheostomies
	10:30-10:45	Coffee break
	10:45-11:15	Caring for a patient with a tracheostomy and documentation
	11:15-12:00	Emergency tracheostomy care
	12:00-12:30	SALT: Communication and swallow
12:20-13:15 Lunch break		
Face-to Face	13:30-16:30	Stations
		1) Emergency scenario – virtual reality
		2) Inner cannula clean
		3) Cuff pressure management
		4) Tapes and dressing change
	16:30-16:45	Debrief and Close

Simulation feedback and cost

The virtual reality headset received positive verbal feedback from participants, who described it as both comfortable and highly immersive. Its ergonomic, close-fitting design facilitated full engagement within the simulated environment. The platform provided immediate performance feedback within the headset interface, displaying real-time results that highlighted correctly executed actions and steps requiring improvement during the simulated management of a blocked or dislodged tracheostomy.

Although not the primary focus of this study, the VR platform also contained additional modules, including front-of-neck emergency access and paediatric tracheostomy obstruction scenarios. The overall cost of delivering the hybrid simulation workshop as part of the study day was approximately £1,200.

Questionnaire and scoring

Knowledge was assessed using an eight-item multiple-choice questionnaire on emergency tracheostomy management (Table 2). The questionnaire was developed by the authors based on the NTSP algorithm [4] and reviewed by two senior ENT consultants for content validity. Each correct response was awarded one point, giving a total possible score of eight. Participants from both groups completed the same questionnaire immediately after training (Day 0) and again after 30 days (Day 30).

**Table 2:** Questionnaire Each correct response was awarded one point (maximum score = 8).

Question Number	Question	Response Options
1	How would you know if you should follow the tracheostomy or laryngectomy emergency algorithm?	Look at the stoma site
		Decide based on the type of tube the patient has in situ
		Look at the completed bedhead sign
2	Which sign is not a red flag that a tracheostomy patient might be in airway distress?	Increased respiratory rate
		Stridor
		Accessory muscle use
		High blood pressure
3	You see a tracheostomy patient in distress, they are breathing. You have called for appropriate help. What is the next step?	Place a suction catheter down the tracheostomy to attempt to unblock it
		Remove the inner tube to replace it
		Check for breathing by looking, listening and feeling at the mouth and tracheostomy
4	When trying to assess the patency of the tracheostomy tube you should remove the speaking valve?	TRUE
		FALSE
5	You have tried to pass a suction catheter and failed. The patient is deteriorating further. What is the next step?	Insert a supraglottic airway device through the mouth
		Insert a oropharyngeal airway through the mouth
		Remove the tracheostomy tube
6	Inserting a cuffed endotracheal tube through the tracheostomy stoma is a method of primary emergency oxygenation?	TRUE
		FALSE
7	If you are ventilating the upper airway using a bag valve mask you need to occlude the tracheostomy stoma?	TRUE
		FALSE
8	When attempting to ventilate via the tracheostomy stoma, which of the following is not an option?	Paediatric face mask
		Laryngeal mask airway (LMA)
		Oropharyngeal airway (Guedel)
		Endotracheal tube

Statistical analysis

Responses were scored and entered into Microsoft Excel (Microsoft Corporation, Redmond, WA, USA) and analysed using GraphPad Prism version 10.1 (GraphPad Software, Boston, MA, USA). Comparative analysis between Day 0 and Day 30 was performed using an independent-samples t-test. 

Normality of data was assessed with the Shapiro-Wilk test, and equality of variances was checked using Levene’s test. Independent samples t-tests were used to compare mean scores between groups at each time point. Mann-Whitney U tests were also conducted to confirm robustness owing to mild non-normality and unequal group sizes. Statistical significance was defined as p < 0.05 (two-tailed).

## Results

A total of 26 participants were included in the study, comprising 17 in the VR group and nine in the non-VR group.

On Day 0, participants in the VR group achieved higher mean knowledge scores (7.24 ± 0.75) compared with those in the non-VR group (6.33 ± 0.87). This difference was statistically significant (t(24) = −2.76, p = 0.0108). At 30 days, the VR group continued to outperform the non-VR group, with mean scores of 6.88 ± 0.93 and 4.67 ± 1.00, respectively (t(24) = −5.64, p < 0.001).

Non-parametric testing using the Mann-Whitney U test confirmed the robustness of these results (Day 0: U = 34.0, p = 0.017; Day 30: U = 8.5, p = 0.0002). The mean difference of approximately 1.5 points between groups corresponded to a large effect size (Cohen’s d ≈ 1.1), indicating a substantial educational benefit of VR-based learning.

Normality testing using the Shapiro-Wilk test confirmed that data were normally distributed in most groups, with mild deviation observed in the VR datasets (p = 0.002-0.024). Levene’s test confirmed equality of variances (p = 0.77 for Day 0; p = 0.83 for Day 30).

A combined summary of group performance, statistical significance, and effect sizes is presented in Table 3.

**Table 3:** Combined Descriptive and Inferential Statistics Comparing Virtual Reality (VR) and Non-VR Groups at Day 0 and Day 30 Data are presented as mean ± standard deviation. Independent-samples t-tests showed significantly higher scores in the VR group at both Day 0 (p = 0.0108) and Day 30 (p < 0.001). Large effect sizes (Cohen’s d ≈ 1.1–1.2) indicate a substantial educational impact of VR-based learning.

Group/Comparison	Day 0 Mean ± SD	Day 30 Mean ± SD	p-value (t-test)	Effect Size (Cohen’s d)	Interpretation				
VR Group	7.24 ± 0.75	6.88 ± 0.93	—	—	—				
Non-VR Group	6.33 ± 0.87	4.67 ± 1.00	—	—	—				
VR vs Non-VR	—	—	Day 0: 0.0108; Day 30: < 0.001	Day 0: 1.1 (large); Day 30: 1.2 (large)	The VR group significantly outperformed the non-VR group at both time points				

**Figure 1:**
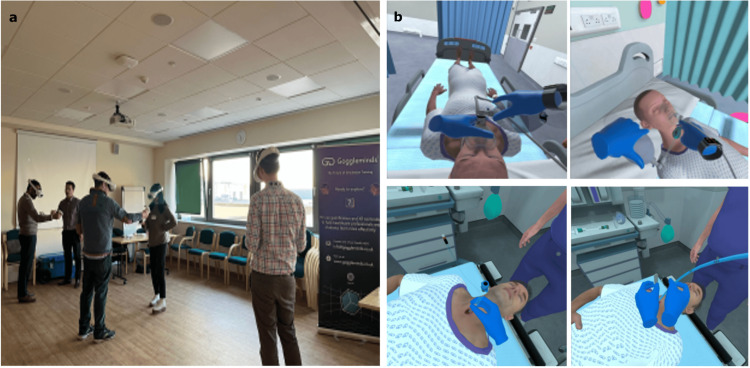
(a) Participant Using the Meta Quest 2 Headset during the Virtual Reality Tracheostomy Simulation. (b) Screenshot of the Simulation Environment Displayed within the Headset. Meta Quest 2 Headset: Meta Platform Technologies, LLC, Menlo Park, United States

An example of the VR tracheostomy simulation using the Meta Quest 2 headset (Meta Platform Technologies, LLC, Menlo Park, United States) is shown in Figure 1.

## Discussion

This study found that VR simulation significantly improved both immediate knowledge acquisition and 30-day retention in tracheostomy education compared with conventional face-to-face teaching. Participants in the VR group achieved higher scores at both assessment points, with large effect sizes (Cohen’s d ≈ 1.1-1.2), indicating a meaningful educational impact. These findings support the role of immersive simulation as an effective adjunct to traditional tracheostomy training.

The statistical analyses confirmed the robustness of these results: equality of variances was maintained, and non-parametric testing produced consistent findings, strengthening confidence in the observed effect.

Comparison with previous studies and validation evidence

VR simulation provides an innovative educational solution to improve clinical performance by replicating realistic scenarios within a high-fidelity virtual environment. This approach offers a safe, repeatable, and engaging platform for healthcare professionals to practise complex or infrequent procedures without risk to patients [4]. Although VR simulation is not a substitute for direct clinical experience, it enables trainees to develop a deeper understanding of anatomy, indications, and procedural techniques through unlimited repetition in a risk-free setting.

In otolaryngology, several validated VR simulators exist for endoscopic sinus surgery, myringotomy, and temporal bone dissection. A systematic review identified that many of these platforms - particularly those focused on sinus and temporal bone procedures - have demonstrated strong validity evidence and potential for inclusion in surgical training curricula [4]. The Voxelman™ TempoSurg® temporal bone simulator, for example, offers realistic haptic feedback and visual detail, allowing users to practise surgical drilling in a lifelike environment [5]. Studies evaluating its content validity have reported positive outcomes [5-7], suggesting that simulation does not necessarily require the highest level of fidelity to be effective, particularly for junior trainees. Repeated practice has been shown to enhance psychomotor learning, especially when supported by appropriate cognitive frameworks.

Construct and predictive validation studies further support the role of VR simulation in improving operative performance. Evidence from the Endoscopic Sinus Surgery Simulator (ES3) indicates that VR-based learning can enhance confidence, instrument handling, and procedural efficiency - findings consistent with results from other surgical specialties [8].

Educational significance and context

The present study extends this growing body of evidence to tracheostomy management - a critical but relatively underexplored area of VR simulation. The immersive and interactive nature of VR likely contributed to improved knowledge retention, consistent with prior research demonstrating that active, scenario-based learning enhances long-term memory and skill consolidation [8].

The Health Education England (HEE) Technology Enhanced Learning framework highlights simulation-based training as a key component of workforce development, promoting safe and effective practice through innovative educational techniques [9]. Our findings align with this national strategy and support broader implementation of VR modules for multidisciplinary teams involved in tracheostomy care.

To build upon these results, we plan to expand the use of VR simulation within our regional training programmes for otolaryngology, oral and maxillofacial surgery, and intensive care trainees. Incorporating VR during induction and ongoing education may help standardise competency in emergency tracheostomy management.

Issleib et al. reported similar outcomes in basic life support education, showing that hybrid or combined VR and traditional approaches yielded the greatest learning gains. Although conventional technical training remains essential, VR was superior in most domains of self-assessment and engagement [10]. Together, these findings suggest that VR functions most effectively as an adjunct to - not a replacement for - traditional methods, reinforcing theoretical and procedural understanding through immersive rehearsal.

Advantages, challenges, and limitations

Despite its clear educational benefits, VR simulation has limitations that must be acknowledged. Some participants in VR-based studies have reported discomfort, dizziness, or nausea, collectively referred to as “motion sickness”, which remains a technological challenge that can only be mitigated through shorter exposure times or increased frame rates [11].

While live simulation offers advantages for teamwork and communication training, it is resource-intensive and logistically demanding. In contrast, VR provides scalable, on-demand access to standardised, reproducible scenarios. This flexibility allows multiple learners to engage concurrently, with integrated performance analytics and immediate feedback, eliminating the need for constant instructor supervision. These features also position VR as a valuable tool for standardised assessment and competency testing [12].

The immersive, repetitive, and interactive qualities of VR are likely key factors behind the superior learning outcomes and retention demonstrated in this study. In contrast, the more passive nature of traditional teaching methods-lacking repetition and real-time feedback-may account for the greater decline in scores observed at 30 days.

Broader implications and future directions

The Royal College of Surgeons of England has highlighted in recent reports that advances in digital and augmented reality technologies must be balanced with cost-effectiveness and equitable access [13]. As technology continues to evolve, identifying educational needs from the perspective of both trainees and trainers will be crucial to ensuring that new tools are implemented efficiently and sustainably.

In response to these challenges, the Association of Surgeons in Training and the RCS England Robotic and Digital Surgery Trainees Group commissioned the Future of Surgery: Technology Enhanced Surgical Training report, which calls for better integration of emerging technologies into structured curricula [13]. Our study contributes to this growing dialogue by providing early evidence of the feasibility and educational value of VR for emergency airway training.

Limitations and conclusions

The primary limitations of this study include the small sample size and the absence of randomisation, which may restrict the generalisability of the findings. Additionally, as a single-centre project evaluating an educational intervention, external validity may be limited. Outcomes were based on knowledge retention rather than direct assessment of procedural skill, which constrains conclusions regarding translation to clinical performance.

Although the results demonstrate encouraging educational outcomes, the study’s design and scale limit broader applicability. Future multicentre, randomised studies with larger participant cohorts are warranted to validate these findings, assess longer-term knowledge and skill retention, and evaluate the impact of VR-based training on real-world clinical performance.

Nonetheless, the findings support the use of VR simulation as a valuable adjunct to traditional tracheostomy education. By providing consistent, immersive, and feedback-driven learning experiences, VR has the potential to standardise training, improve clinician confidence, and enhance patient safety in airway management.

## Conclusions

VR simulation represents a valuable, innovative adjunct to traditional tracheostomy training. This study demonstrated that VR-based education significantly improved both immediate knowledge acquisition and 30-day retention when compared with standard teaching methods. The immersive, interactive, and feedback-driven nature of VR appears to foster deeper learning, enhance confidence, and encourage repeated deliberate practice. As simulation technology continues to evolve, its integration into otolaryngology and multidisciplinary airway management programmes may help to standardise training and improve patient safety across healthcare settings.
